# A personalized decision aid for prostate cancer shared decision making

**DOI:** 10.1186/s12911-021-01732-2

**Published:** 2021-12-31

**Authors:** Hilary P. Bagshaw, Alejandro Martinez, Nastaran Heidari, David Scheinker, Alan Pollack, Radka Stoyanova, Eric Horwitz, Gerard Morton, Amar U. Kishan, Mark K. Buyyounouski

**Affiliations:** 1 Stanford University Radiation Oncology, Stanford, CA USA; 2 School of Engineering, Stanford University, Stanford, CA USA; 3 University of Miami Sylvester Comprehensive Cancer Center, University of Miami Health System, Miami, FL USA; 4 Fox Chase Cancer Center, Philadelphia, PA USA; 5 Sunnybrook Odette Cancer Centre, Toronto, ON Canada; 6 University of California, Los Angeles, Los Angeles, CA USA

**Keywords:** Decision aid, Shared decision making, Prostate cancer, Personalized medicine, Radiation therapy preference based decisions

## Abstract

**Background:**

A shared decision-making model is preferred for engaging prostate cancer patients in treatment decisions. However, the process of assessing an individual’s preferences and values is challenging and not formalized. The purpose of this study is to develop an automated decision aid for patient-centric treatment decision-making using decision analysis, preference thresholds and value elicitations to maximize the compatibility between a patient’s treatment expectations and outcome.

**Methods:**

A template for patient-centric medical decision-making was constructed. The inputs included prostate cancer risk group, pre-treatment health state, treatment alternatives (primarily focused on radiation in this model), side effects (erectile dysfunction, urinary incontinence, nocturia and bowel incontinence), and treatment success (5-year freedom from biochemical failure). A linear additive value function was used to combine the values for each attribute (side effects, success and the alternatives) into a value for all prospects. The patient-reported toxicity probabilities were derived from phase II and III trials. The probabilities are conditioned on the starting state for each of the side effects. Toxicity matrices for erectile dysfunction, urinary incontinence, nocturia and bowel incontinence were created for the treatment alternatives. Toxicity probability thresholds were obtained by identifying the patient’s maximum acceptable threshold for each of the side effects. Results are represented as a visual. R and Rstudio were used to perform analyses, and R Shiny for application creation.

**Results:**

We developed a web-based decision aid. Based on preliminary use of the application, every treatment alternative could be the best choice for a decision maker with a particular set of preferences. This result implies that no treatment has determinist dominance over the remaining treatments and that a preference-based approach can help patients through their decision-making process, potentially affecting compliance with treatment, tolerance of side effects and satisfaction with the decision.

**Conclusions:**

We present a unique patient-centric prostate cancer treatment decision aid that systematically assesses and incorporates a patient’s preferences and values to rank treatment options by likelihood of achieving the preferred outcome. This application enables the practice and study of personalized medicine. This model can be expanded to include additional inputs, such as genomics, as well as competing, concurrent or sequential therapies.

## Background

Prostate cancer is the second most common cancer diagnosed in the United States [1], and patients have multiple treatment options with similar efficacy. While physicians see this as opportunity for shared decision making (SDM), patients often feel overwhelmed with anticipated regret. Without proper understanding about treatment modalities, outcomes and risk–benefit profiles, these “preference sensitive” [2] decisions are stressful for patients, which increase rather than decrease the burden of prostate cancer.

Decision aids (DA) are effective tools that can be utilized in SDM to educate patients. They have been shown to improve patient-provider communication, knowledge and accuracy of perceptions, decrease decisional conflict, indecision and patients feeling uninformed [2, 3]. Some evidence even shows that patient compliance is improved with the utilization of such DAs [4]. Without guidance, physicians may deem SDM burdensome, putting them behind in a busy clinic and creating more work, however the use of DAs has a positive effect on the patient-provider relationship and patients using DAs are either as satisfied or more satisfied with the process compared to those that do not use DAs [2]. While there are some DAs for prostate cancer patients, personalization is lacking, leaving patients unclear of how these treatments will specifically affect them. Misuse of DAs or those of poor quality can also contribute to negatively to patient decision making, increasing stress, or posing challenges for implementation [3, 5]. DAs should be created according to the International Patient Decision Aid Standards (IPDAS) criteria to ensure quality and optimal utilization [6].

The prostate cancer community needs a data-driven DA that both assesses and incorporates individual patient preferences, such that patients and physicians feel confident using it for SDM. We developed a novel DA for this population, which allows men newly diagnosed with prostate cancer, who have multiple equivalent treatment options, to evaluate these with their providers as part of SDM, in a personalized manner in the context of their pre-treatment health state.

## Methods

The development of this DA was completed under an institutional approved IRB. A group of experts in the fields of genitourinary radiation oncology and decision analysis were assembled. Following multiple focus group meetings and joint observations during prostate cancer consultations in the radiation oncology clinic over a 3-month period, the group of radiation oncologists and decision analysis experts created a template for the DA. We considered the IPDAS criteria [6] during creation and focused on the treatment modalities active surveillance and radiation therapy. This template included the following inputs: National Comprehensive Cancer Network (NCCN) risk group, patient pre-treatment health state, active surveillance or radiation treatment options according to risk group, anticipated side effects and anticipated treatment success.

### Risk group

For prostate cancer patients, risk grouping is used to determine appropriate treatment options. Risk grouping was defined as per the risk stratification defined by the NCCN [7]. We chose to simplify the risk groups into three broad categories (low risk, intermediate risk, and high risk), as much of the outcomes data is reported this way. The risk categorization and definitions of each NCCN risk group is displayed in Table 1.

**Table 1:** National Comprehensive Cancer Network (NCCN) risk grouping for prostate cancer [7]

Risk category for DA	NCCN risk group	Clinical and pathologic features
Low	Very low	*All of the following features*
		cT1c
		Grade Group 1
		PSA < 10 ng/mL
		< 3 positive biopsy cores, < *50% in each core*
	Low	*All of the following but doesn’t qualify for very low risk*
		cT1c-T2a
		Grade Group 1
		PSA < 10 ng/mL
Intermediate	Favorable intermediate	*All of the following features*
		1 intermediate risk factor (cT2b-T2c, Grade Group 2, PSA 10-20 ng/mL)
		Grade Group 1 or 2
		< 50% biopsy cores positive
	Unfavorable intermediate	*One or more of the following*
		2 or 3 intermediate risk factors (cT2b-T2c, Grade Group 2 or 3, PSA 10-20 ng/mL)
		Grade Group 3
		≥ 50% biopsy cores positive
High	High	*No very high risk features and exactly one high risk feature*
		cT3a
		Grade Group 4 or 5
		PSA > 20 ng/mL
	Very High	*At least one of the following*
		cT3b-T4
		Primary Gleason pattern 5
		2 or 3 high risk features
		> 4 cores with Grade Group 4 or 5

*DA* decision aid, *NCCN* National Comprehensive Cancer Network, *c[cT1c]* clinical stage, *T1c* tumor identified by needle biopsy in one or both sides, *T2a* tumor involves one-half of one side or less, *T2b* tumor involves more than one-half of one side, *T2c* tumor involves both sides, *T3a* extraprostatic extension, *T3b* seminal vesicle invasion, *T4* tumor is fixed or invades adjacent structures

### Treatment alternatives and success

This DA is focused on radiation treatment options and thus alternatives included active surveillance (AS), stereotactic body radiation therapy (SBRT), external beam radiation therapy (EBRT) delivered with intensity modulated radiation therapy in both standard fractionation and hypofractionated courses, EBRT + high dose rate brachytherapy (HDR), EBRT + androgen deprivation therapy (ADT), and EBRT + HDR + ADT. While surgery is another accepted alternative, it was not included in this DA due to lack of toxicity input data.

The group of experts defined appropriate radiation treatment options according to risk group, in accordance with recommendations from the NCCN [7]. Expected outcomes after radiation therapy treatments were defined by risk groups in terms of 5-year freedom from biochemical failure based off expert knowledge and literature review.

### Pre-treatment health state

Four common radiation related side effects were selected to define the patient’s pre-treatment health state based on the focus groups and clinic observations. These included erectile dysfunction (ED), urinary incontinence, urinary frequency/nocturia and bowel incontinence. The questions to define the pre-treatment health state were taken from the Expanded Prostate Cancer Index Composite (EPIC) [8] validated questionnaire and the International Prostate Symptom Score (IPSS) [9]. In the DA, the patient is instructed to relate the questions to the past 4 weeks. The questions are defined as follows. For ED, “…what percentage of the time that you wanted to have an erection, were you able to achieve one”, for urinary incontinence, “…how often have you had uncontrolled leakage of urine”, for urinary frequency (nocturia), “…how many times have you woken up to go to the bathroom after you went to bed”, and for bowel incontinence “…how often have you had uncontrolled leakage of stool or feces”.

### Anticipated side effects

Common radiation related side effects including ED, urinary incontinence, urinary frequency/nocturia and bowel incontinence were selected as above. Rather than defining toxicities as a certain predicted percent, as taken from published literature in our DA, the predicted chance of a certain toxicity was defined as a probability based off patient-reported outcomes (PROs) data after collaboration with three other institutions from clinical trials.

Following IRB approval, we compiled pre-treatment EPIC data and patient-reported post-treatment EPIC data from three different institutions in order to define toxicity tables. The toxicity tables related the pre-treatment health state (EPIC response) to the probability of a certain post-treatment health state (EPIC response) for each radiation modality. The PROs toxicity probabilities for EBRT and EBRT + ADT were derived from the Fox Chase Phase III hypofractionation trial [10]. The probabilities for EBRT + HDR were derived from patients treated at Odette Cancer Centre, Sunnybrook Health Sciences Centre [11]. The probabilities for SBRT were derived from patients treated at The University of California at Los Angeles (UCLA) [12]. As AS does not involve active treatment, we elected not to attribute side effects to this option, however we discuss limitations of this in the discussion section.

R and R Studio were used for statistical analysis and R Shiny was used for web-application creation.

## Results

### Defining value function and preference thresholds

We use the concept of preference thresholds (PT) [10] to reduce the problem in size and personalize it to each decision maker. PTs are used to calculate probability that a side effect is worse than a patient set threshold, and to reduce alternatives and simplify the creation of a value function [10]. We used a linear additive value function by combining value driving attributes (uncertainties or anticipated side effects and treatment alternatives). The specifics of the value functions, probabilities and preference thresholds have been previously reported [13]. Following creation of these value functions and preference thresholds, toxicity tables using PROs (see *Toxicity Tables* below) from different institutional studies were utilized to define the uncertainties and create the backbone of the DA.

**Fig. 1:**
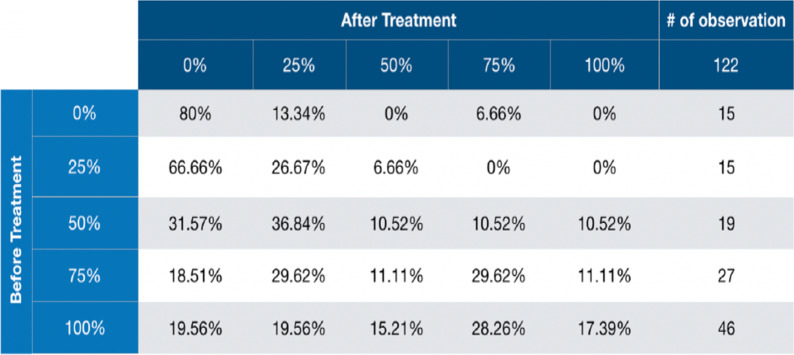
Erectile dysfunction toxicity table. Changes in erectile function are displayed comparing pre-treatment erectile function and post-treatment erectile function as percentage of potency. The percentage of patients with 0, 25%, 50%, 75% or 100% erectile function post-treatment is displayed. For example, for the 46 patients that had 100% potency pre-treatment, only 17.39% retained 100% potency post treatment

After obtaining PROs data from Fox Chase, Sunnybrook and UCLA (see *Methods*), a correlation analysis was performed to understand which features could be used to predict post-treatment side effect levels. This analysis showed us that the best predictor of post treatment state of side effects is the pretreatment state for each patient. We used this information to calculate toxicity transition probability tables. These tables relate the pre-treatment health with probabilities of transitioning to every post-treatment health. These tables were constructed for the common side effects ED, urinary incontinence, urinary frequency/nocturia and bowel incontinence, and for each treatment alternative; SBRT, EBRT, EBRT + HDR, EBRT + ADT, and EBRT + HDR + ADT using approximately 150 patients per treatment approach. As an example, Fig. 1 displays a toxicity probability table for ED. In this example, patients who had perfect erectile function pre-treatment (row 100%) tended to see a decline in their erections post-treatment (column 0–75%) and only 17% of them retained their excellent erectile function post-treatment. The conditional probabilities of achieving a post treatment state were calculated using a frequentist count for each possible starting state for each of the side effects [13].

### Web-based decision aid

Finally, a web-based application was created that can be used to define preference thresholds, assign value and evaluate alternative treatment options.

**Fig. 2:**
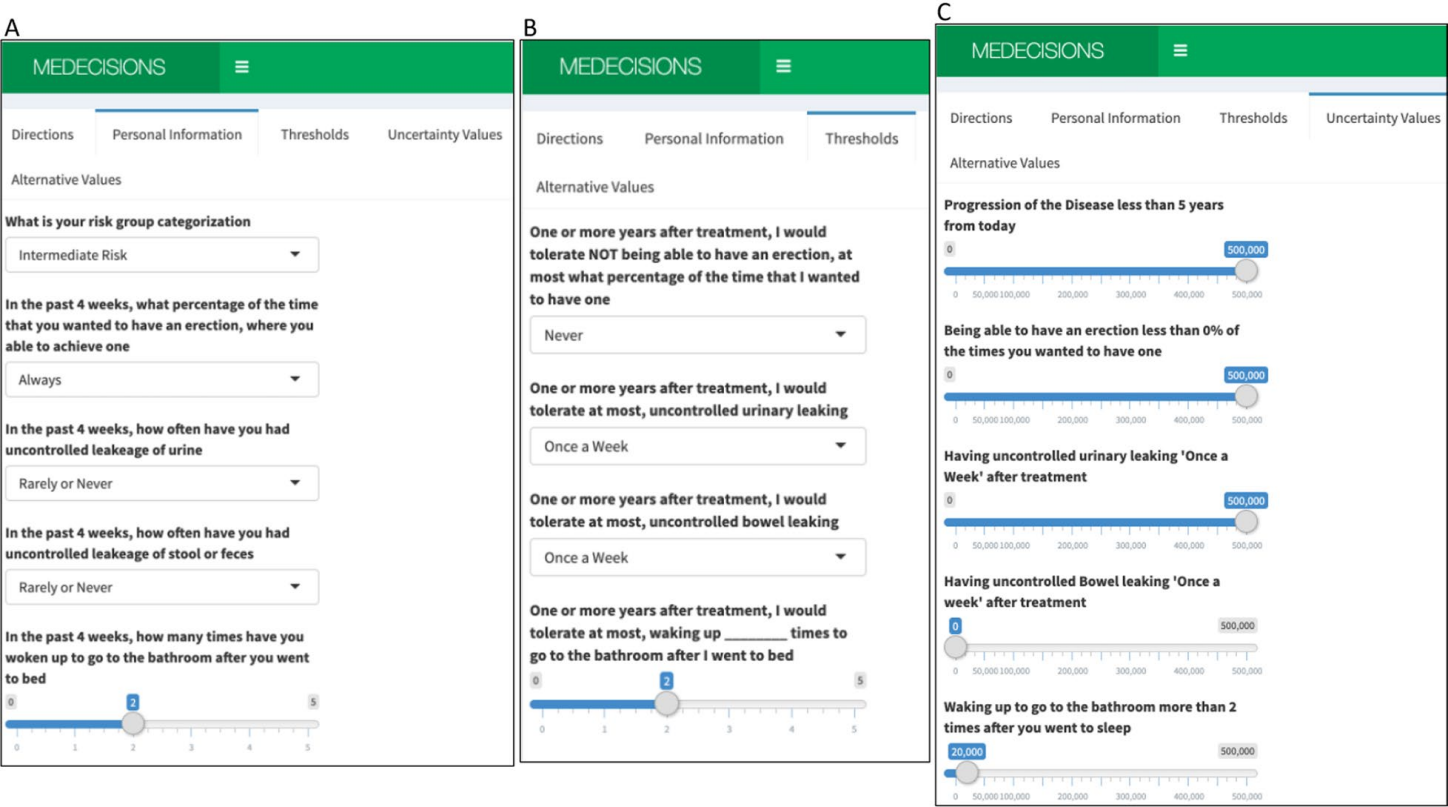
The decision aid online information entry pages. *This web-based aid is our own independent work, not taken from elsewhere*. **A** Patient information page, where patients enter their risk group and current health state. **B** Threshold page, where patients enter their personal thresholds. **C** Uncertainty value page, where patients enter how much they would be willing to pay to avoid a certain situation

The DA allows patients to input personal information about their stage of cancer and pre-treatment health state (Fig. 2A). Next, patients are asked their maximum acceptable threshold for a particular side effect, “for each of the following side effects, please indicate your maximum tolerance, once all side effects have stabilized 1 + year after treatment” (Fig. 2B) with questions taken from the EPIC. Next, patients assess the value of living with a side effect above their acceptable threshold, and are asked how much “would we have to pay you to accept this health state for the rest of your life?” (Fig. 2C), and the health state is mirrored from the Threshold page (Fig. 2B). For example, in Fig. 2B the patient selected they would tolerate waking up 2 times to go to the bathroom after going to bed. This threshold is then translated into Fig. 2C, and the patient is asked how much we would have to pay them to accept always waking up more than 2 times at night to go to the bathroom.

**Fig. 3:**
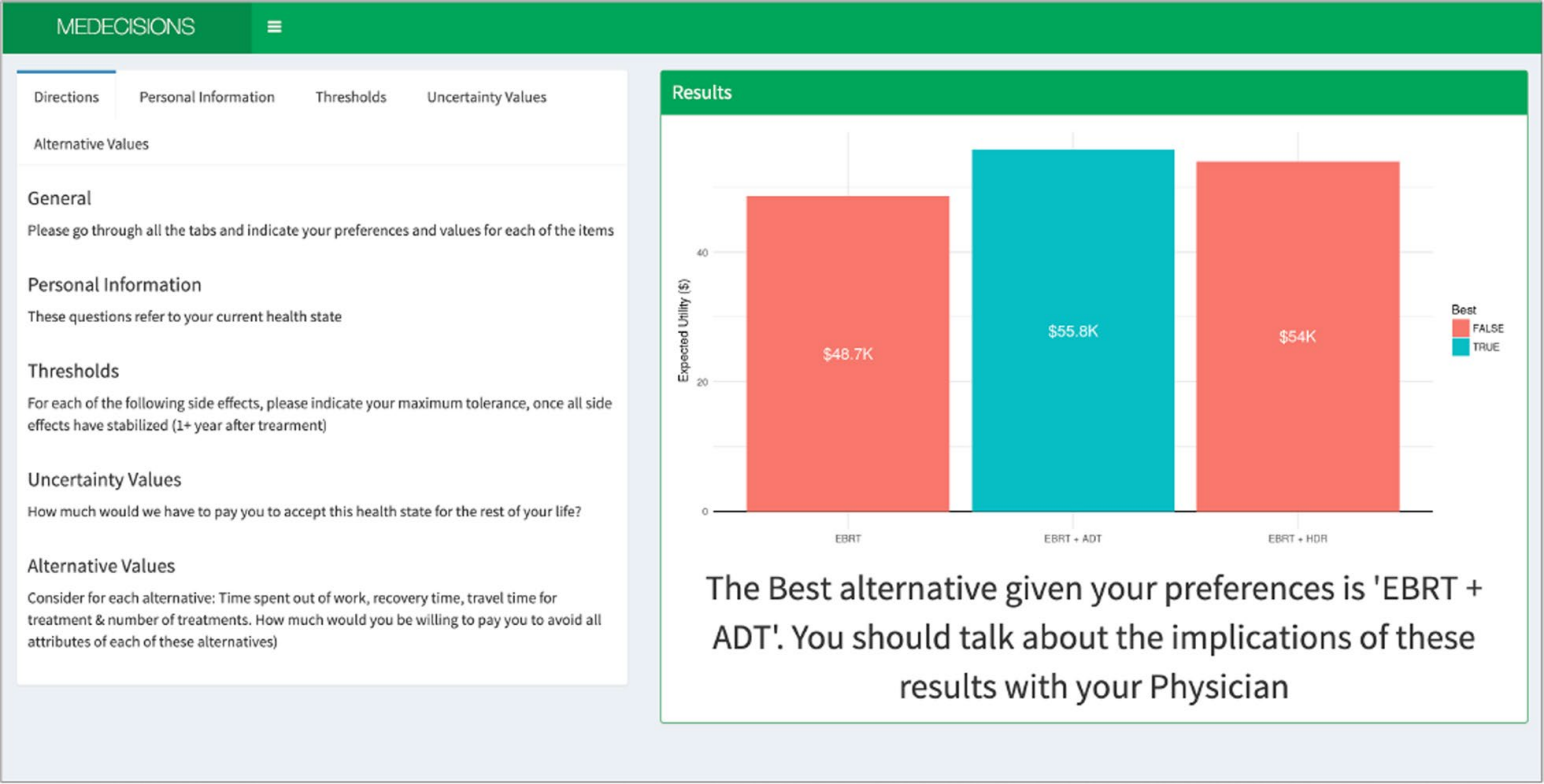
The Decision Aid online results page. *This web-based aid is our own independent work, not taken from elsewhere*. Directions are displayed on the left-hand side along with information entry pages if the user clicks on different headings (as showing in Fig. 2). The results are displayed on the right-hand side of the screen, which adjust in real time based on the inputs on the left-hand side. This version uses willingness to pay to display results, thus depicted as monetary value. [*EBRT* external beam radiation therapy, *ADT* androgen deprivation therapy, *HDR* high dose rate brachytherapy]

Patients can also add value to treatment alternatives based on non-medical criteria when asked, “how much would we have to pay you to endure all attributes of each of these alternatives (Number of visits, time at the hospital, etc.)”. A patient with low-risk prostate cancer may prefer to minimize the number of trips to the facility, thus could place more value to SBRT compared to EBRT. Results are represented as a visual (Fig. 3), with the best alternative represented in teal, and other alternatives represented in red. We use willingness to get paid in the assessment of preferences because dollars represent a common medium that is easily understood by every patient regardless of their education level. The questions are about willingness to get paid to endure rather than willingness to pay, because we want to avoid patients thinking of the balance in their accounts as a limiting factor. In these cases, patients will express a much higher amount to endure outcomes that they consider worse. Assessments of this kind done on Likert scales are much harder to perform correctly since the difference between a “3” and a “4” in a Likert scale is not clearly defined for everyone, whilst the difference between $50 and $100 is exact and equal for all patients. Results are thus represented in dollar amounts attributed to the expected value that the decision maker places on each alternative, after considering all the potential side effects and treatment specific characteristics. Importantly, the results are displayed on the right-hand side of the screen as the patient enters inputs for their preference thresholds, uncertainty values and alternative values and as the inputs change, the patient can see the results change in real time (Fig. 3). This allows patients to toggle between preferences, values and alternatives and evaluate the different outcomes based on different inputs.

Based on preliminary use of the application, it has been observed that every treatment alternative could be the best choice for a decision maker with a particular set of preferences. This result implies that no treatment has deterministic dominance over the remaining treatments and that a preference-based approach can help patients through their decision-making process.

## Discussion

We developed a novel web-based DA, uniquely designed to allow patients with prostate cancer to evaluate different treatment modalities, outcomes and side effects based on their personal preferences and pre-treatment health state. While other DAs exist, there are few and they do not relate risk of toxicities to the specific patient. The IPDAS criteria for a quality DA recommends methods for “clarifying and expressing patients’ values” [6], which this DA specifically focuses on. As every patient has a different pre-treatment health state, discussion of toxicities in the context of the risk to that specific patient is imperative. For example, if patient A already has erectile dysfunction, they likely will not be concerned about further risk of ED and thus not attribute much value to this uncertainty, whereas patient B may have perfect erectile function and place more value on preservation of sexual function compared to even treatment success.

This DA provides a missing piece to the good decision model. In the world of decision analysis, experts classically describe the quality of one’s decision based upon balance of the three-legged stool, representing three pillars of information the decision maker needs for a quality decision; information, alternatives and personal preferences [14]. Physicians are well versed at providing patients with information about their diagnosis, alternative treatment options, probability of a certain outcome (for example cancer cure) and side effect profiles. These skills are entrenched in the training of physicians practicing evidence-based medicine, providing data behind each decision and recommendation. It is the third pillar, patient preferences, which physicians struggle with most because the physician has a hard time providing personalized probabilities and of course, physicians cannot provide patients with information about their personal preferences. In order to elicit patient preferences, physicians must change their mindset to “diagnosing [patient] preferences” [4] in order to strengthen the third pillar. They must enable patients to participate in their own decision making by helping them define these preferences, thus balancing the three pillars of decision analysis, with the result being a quality decision.

This DA will fill a gap, as most DAs for SDM in prostate cancer exist as booklets or online education sites [15], with general information about treatment options and side effects, focused on usability [16]. Even though physicians have been encouraged to participate in SDM and “patient-centered-care” since the 1980s [4], patients still report that physicians make treatment related decisions without involving them [17]. The American Urological Association, American Society for Radiation Oncology and Society for Urologic Oncology (AUA/ASTRO/SUO) recognize the importance of SDM, recommending clinicians utilize SDM for patients diagnosed with prostate cancer; however, these organizations provide no recommendations on how exactly to implement SDM [18]. Importantly, the Centers for Medicare & Medicaid Services (CMS) requires SDM in certain situations, for example patients undergoing low dose CT screening for lung cancer must undergo counseling and SDM, including the use of a decision aid [19]. The CMS Oncology Care Model also discusses SDM [20], and perhaps decision aids will permeate oncologic care reimbursement in the future. As DAs become more prevalent in oncologic care models, we need to ensure quality and that they are in accordance with the IPDAS [6].

This personalized DA makes a problem with potentially multiple outcomes a binary one, thus reducing the burden on the decision-maker, the patient. Due to the robust PROs data published and utilized in this DA for radiation treatment modalities, we can individualize the results based on pre-treatment health state and personal preferences to post-treatment side effects or preferences regarding alternative treatments. This ultimately allows for improved SDM as patients can define their thresholds, evaluate treatment outcomes and toxicities based on their health state and preference thresholds. Importantly, this DA also allows patients to change or adjust their preferences and thresholds, visualizing how that may change the treatment recommendation in real time. Those who may not be sure about their preferences have time to ponder, adjust, and digest the information with a provider or in private. This DA is not meant to replace consultation with a specialist, rather enhance that discussion, facilitate SDM, and empower patients with knowledge.

Furthermore, this DA, or the methodology employed, could be embedded in existing DAs, customized on an institutional basis, or scaled to be more comprehensive. A limitation of this DA is its lack of surgical and brachytherapy monotherapy data. We are working to add more PROs data from other collaborators, which will make the data in the current DA more robust and allow for additional treatment alternatives to be included. In addition, in this model, no side effects were attributed to active surveillance, however a more accurate depiction of that treatment option would be to include PROs for men managed this way as they also can have urinary and erectile side effects with aging [21]. We call for collaboration within the prostate cancer community, as more data will make this DA an even more powerful tool for all our patients.

Future directions include implementation of this DA in our radiation oncology consultation appointments. Through an IRB approved project, we will allow patients to use the DA and elicit feedback via a survey as well as one-on-one interviews, allowing for adjustments to this DA as we understand what is most important to patients and as recommended by the IPDAS [6]. Other groups have reported challenges with implementation of DAs, at both the clinician and system level [3], however we are dedicated to bringing this DA to life in order to strengthen the quality of the decisions prostate cancer patients make about their treatment.

## Conclusions

In conclusion, we developed a novel personalized, patient-centric systematic quantitative and visual approach to prostate cancer treatment decision making that we hope to make widely available to patients and physicians alike to lessen the burden of prostate cancer.

## Data Availability

The datasets used and/or analyzed during the current study are available from the corresponding author on reasonable request.

## Abbreviations

DA: Decision aids
IPDAS: International Patient Decision Aid Standards
SDM: Shared decision making
IRB: Institutional Review Board
NCCN: National Comprehensive Cancer Network
PSA: Prostate specific antigen
AS: Active surveillance
SBRT: Stereotactic body radiation therapy
EBRT: External beam radiation therapy
HDR: High dose rate
ADT: Androgen deprivation therapy
ED: Erectile dysfunction
EPIC: Expanded Prostate Cancer Index Composite
IPSS: International Prostate Symptom Score
PROs: Patient-reported outcomes
UCLA: University of California at Los Angeles
AUA: American Urological Association
ASTRO: American Society for Radiation Oncology
SUO: Society of Urologic Oncology
